# Cardiorespiratory fitness profile of volunteer firefighters during intermittent incremental exercise: A cross-sectional study

**DOI:** 10.1371/journal.pone.0353529

**Published:** 2026-07-23

**Authors:** Denisse Bustos, Filipa Cardoso, Manoel J. Rios, Ricardo Cardoso, Diogo D. Carvalho, Joana Guedes, José Torres Costa, Mário Vaz, J. Santos Baptista, Ricardo J. Fernandes

**Affiliations:** 1 Associate Laboratory of Energy, Transports and Aeronautics, LAETA, Faculty of Engineering, University of Porto, Porto, Portugal; 2 Centre of Research, Education, Innovation and Intervention in Sport, CIFI2D, Faculty of Sport, University of Porto, Porto, Portugal; 3 Porto Biomechanics Laboratory, LABIOMEP-UP, Faculty of Sport, University of Porto, Porto, Portugal; 4 Piaget Research Center for Ecological Human Development, Higher School of Sport and Education, Jean Piaget Polytechnic Institute of the North, Vila Nova de Gaia, Portugal; 5 Research Center in Physical Activity, Health and Leisure, CIAFEL, Faculty of Sport, University of Porto, Porto, Portugal; 6 Laboratory for Integrative and Translational Research in Population Health, ITR, Porto, Portugal; 7 Associate Laboratory of Energy, Transports and Aeronautics, LAETA, Faculty of Medicine, University of Porto, Porto, Portugal; Ruhr University Bochum, GERMANY

## Abstract

**Purpose:**

We aimed to assess the physiological profile of male volunteer firefighters at a broad spectrum of exercise intensities to determine whether their cardiorespiratory fitness level is adequate to meet the physiological demands of their occupational activities.

**Methods:**

Thirty-one male volunteer firefighters performed an intermittent incremental treadmill running exercise (with 4 min steps, 1 km·h^−1^ increments and 30 s rest periods). Cardiorespiratory variables were continuously monitored and blood lactate concentrations were obtained at baseline, throughout rest periods and post-exercise. The study followed a cross-sectional laboratory-based assessment, in accordance with the STROBE guidelines for cross-sectional studies.

**Results:**

The increase in running velocity resulted in progressively higher values from low to severe exercise intensities in oxygen uptake (from 23.6 ± 7.5 to 44.1 ± 7.6 mL·kg^−1^·min^−1^; *p* < 0.001; η_p_^2^ = 0.560), heart rate (from 133 ± 24 to 185 ± 14 bpm; *p* < 0.001; η_p_^2^ = 0.459) and energy expenditure (from 127 ± 50 to 303 ± 51 kJ; *p* < 0.001; η_p_^2^ = 0.654). Aerobic energy contributions progressively declined (94–83%), while anaerobic contributions increased (6–17%) across the incremental intensity domains. Inverse associations were reported between heart rate and body mass index at heavy (r = −0.38; *p* = 0.017) and severe (r = −0.34; *p* = 0.028) intensities, and with fat mass at heavy intensity (r = −0.32; *p* = 0.037).

**Conclusions:**

Findings evidenced a moderate to strong effect of exercise intensities on most assessed variables, and approximately half of the participants (~52%) did not meet the age-specific oxygen uptake threshold recommended by widely referenced National Fire Protection Association (NFPA-USA) guidelines for performing their professional duties. Given the firefighters’ high physiological demands, they should follow structured training interventions and receive targeted nutritional guidance to improve their cardiorespiratory fitness while ensuring optimal performance in the field.

## Introduction

Tactical professionals such as firefighters, law enforcement officers and military personnel, operate in physically demanding, high-risk environments marked by extended shifts, harsh climates, sleep deprivation and suboptimal nutrition, which collectively increase the risks of stress, overexertion and injury [1–3]. Furthermore, health issues such as cardiovascular disease, reduced pulmonary function and hypertension, are prevalent among these professionals, with these conditions posing major concerns due to the nature of their occupational duties [4–6]. Firefighting duties encompass fire suppression, rescue operations, hazardous material incident response and emergency medical interventions [7]. As their roles require strenuous physical performance, even short-duration tasks can increase oxygen uptake (VO_2_) and heart rate (HR) at near-maximal levels, resulting in substantial energy expenditure and intense perceived exertion [7–9]. As a result, fireground operations generally fall within the heavy and severe intensity domains, while recovery after an emergency and routine activities are equivalent to low and moderate intensities (Table 1) [10–12].

**Table 1:** Correspondence between common firefighting tasks and exercise intensity domains.

Firefighting task	Frequent operational context	Equivalent intensity domains
Forcible entry	Sledgehammer or halligan use, often following stair climb or hose advance	Severe
Stair or ladder climb while carrying a high-rise pack	High-rise fire response	Heavy–Severe
Hose dragging and advancement	Interior structural firefighting	Heavy–Severe
Victim rescue and dragging	Fireground search and rescue	Heavy
Overhaul and salvage	Post-suppression operations	Moderate–Heavy
Equipment preparation and staging	Pre-incident staging	Low–Moderate
Recovery following emergency calls	Post-incident rest period	Low
Administrative and fire brigade duties	Non-operational tasks	Low

Given their essential role in emergency preparedness and the physical demands of their occupation, preventive and interventional measures should be implemented before unacceptable risk levels are reached [13]. Extensive evidence demonstrates that higher physical fitness provides protection against major chronic diseases and enhances occupational performance by improving endurance, accelerating recovery and delaying fatigue onset [2,4,14]. Therefore, physical fitness assessments can provide valuable insights into general health and well-being, ensuring job-task capability and the implementation of informed preventive actions [2,15,16]. Although fitness standards exist for firefighters, they are inconsistently applied, particularly among volunteer populations, due to operational variability [16,17]. The most widespread guidelines are from the National Fire Protection Association (NFPA) standard NFPA 1582 from the USA, in which a maximal VO_2_ of 42 mL·kg^−1^·min^−1^ is advised as the minimum threshold to ensure firefighters can safely engage in job-related tasks [18].

However, the latest amendment to this standard and its consolidated version in the NFPA 1580 standard (last updated in 2025) have included age and gender-based threshold levels for evaluating cardiorespiratory fitness [18,19]. The NFPA 1580 is part of NFPA’s Emergency Response and Responder Safety standards consolidation plan and integrates elements formerly addressed across multiple NFPA documents [19]. In the UK, the role-related minimum cardiorespiratory fitness standard for operational firefighters is > 42 mL·kg^−1^·min^−1^, with fire brigades considering individuals with maximal VO_2_ values between 36–42 mL·kg^−1^·min^−1^ unfit for duty and requiring fitness training [17]. Previous research has also reported that firefighters may require values > 44 mL·kg^−1^·min^−1^ to ensure optimal performance during their most strenuous tasks [11,20]. However, in many regions, volunteer firefighters are exempt from mandatory fitness evaluations, unlike their career counterparts, hindering accurate assessment and tailored training prescriptions [2,21].

Although occupational stress and risk exposure are common to all firefighting personnel, training, physical and mental conditions vary among individuals, with this heterogeneity being particularly evident among volunteer firefighters [22,23]. Most have a main external occupation and perform firefighting duties outside their working hours, with adequate physical fitness being essential to manage these demanding workloads [21]. However, most of these groups are not engaged in regular training programs or periodically evaluated regarding their physical fitness [2,21]. In addition, there is evidence that declines in job performance among firefighters are related to the function of both anaerobic and aerobic energy systems, which has not been comprehensively addressed among volunteer groups [12,24]. The energy release rate is influenced by effort intensity and duration, providing valuable information for adjusting training programs [25,26]. Among some typical fire ground duties, high-intensity short-duration efforts (e.g., hose line advance and forcible entry) are primarily supported by the anaerobic energy system, while moderate (e.g., load carriage and victims rescue) and low-intensity longer tasks (e.g., crawling, searching and salvage) are mainly sustained by the aerobic metabolism [12,25,27]. Furthermore, body composition may influence their ability to perform physically demanding tasks, with the literature indicating that certain related variables (e.g., high fat mass and body mass index) affect cardiorespiratory fitness and contribute to earlier fatigue onset [11,17].

Considering the challenges associated with this profession and the pivotal importance of good physical condition for their specialised operations, specific guidelines and individualised assessments should be strongly recommended [15,16]. While research on firefighter and other first responders fitness exists [7,16,27,28], few studies focus on volunteer firefighters [21–23,29]. Furthermore, they are based on exercise protocols that may not be suitable for these professionals and their individual profiles [11,30,31]. A treadmill protocol conducted under controlled conditions reduces the constraints associated with field measurements while reliably eliciting comparable physiological responses [30–32]. Its alignment with normative recommendations, together with its practicality for periodic assessment of firefighters’ physical condition, supports its relevance [33]. Thus, the current study aimed to evaluate and characterise the physiological profile of male volunteer firefighters across exercise intensities, using an individualised running protocol. We expected that their cardiorespiratory fitness would meet the NFPA 1580 recommendations [19], and hypothesised that significant associations would emerge between cardiorespiratory fitness and body composition metrics.

## Methods

### Participants

Active volunteer firefighters from two local fire brigades were invited, and thirty-one male firefighters agreed to participate in the study (age: 32.3 ± 10.5 years, body mass: 78.0 ± 11.9 kg, body height: 174.5 ± 6.0 cm, body mass index: 25.7 ± 4.2 kg·m^−^^2^ and body fat percentage: 19.7 ± 7.6%). Recruitment was conducted from May 10 to July 1, 2022, through convenience sampling. Participants reported performing vigorous physical activity ≥ three times/week (no inclusion criterion was established regarding physical fitness) and had no history of cardiopulmonary or musculoskeletal conditions. Physical activity was obtained by questionnaire (the International Physical Activity Questionnaire), and no objective monitoring (e.g., training logs or wearable data) was performed. Measurements were collected under standardised pre-test conditions, instructing participants to abstain from strenuous exercise and alcohol consumption in the 48 h prior to the test, as well as avoid caffeine for 6 h before testing and arrive hydrated. All eligible firefighters from the two brigades were invited to participate. However, information regarding refusals or exclusions was not systematically recorded. The research was approved by the Ethics Committee of the University of Porto (Report 106/CEUP/2021, approved on April 13, 2021) and individuals were informed about the purpose, experimental procedures, potential benefits and risks of their participation, providing their previous written individual consent in accordance with the Declaration of Helsinki. The study design followed a cross-sectional laboratory-based assessment.

### Experimental protocol

**Fig 1:**
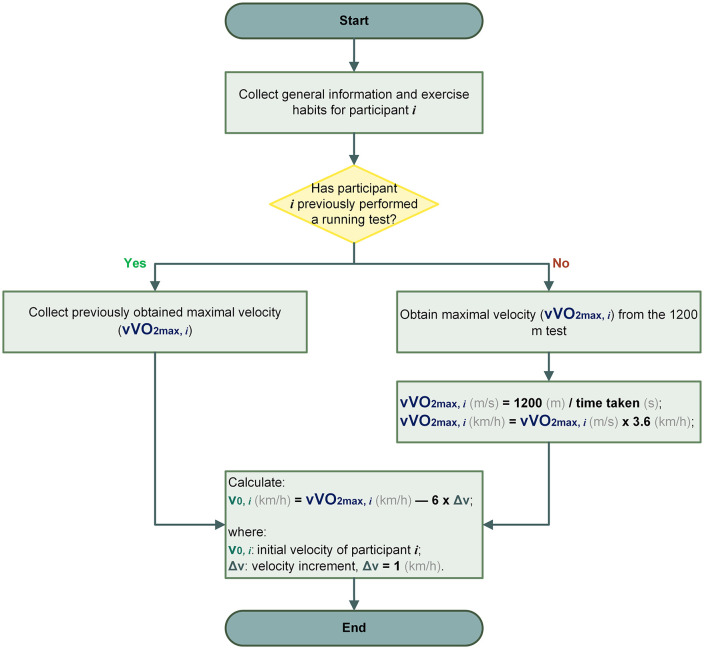
Decision flow algorithm for determining participant initial running velocity.

Body mass, body fat, fat-free mass and body mass index from participants were determined using an InBody 270 bioimpedance scale (InBody Co. Ltd., Cerritos, CA, USA; ± 3% accuracy), and height was measured using a Seca 220 stadiometer (Seca GmbH, Hamburg, Germany; ± 5 mm accuracy). Participants wearing standardised light clothing (~0.3 clo), completed an intermittent individualised incremental running protocol to volitional exhaustion (with 4 min steps, 1 km·h^−1^ increments and 30 s rest periods in-between) on a treadmill (T2100 treadmill; GE, Boston, MA, USA) in a climatic chamber (FITOCLIMA 25000EC20; Aralab, Rio de Mouro, Portugal) [32,34] maintained at thermoneutral conditions (24 °C; 50% RH) [33,34]. The velocities for each participant’s last step were determined based on their best individual 1200 m performance at the time of data collection or on their experience from previous tests (27 and 4 participants, respectively). The final-stage velocity was determined as the mean running velocity achieved during the 1200 m test (distance/time) [35]. For the second case, the final velocity was estimated based on prior testing experience and adjusted to ensure volitional exhaustion within the expected test duration [32,36]. The six velocity increments were then subtracted to define the subsequent step paces [32,34] (Fig 1).

**Fig 2:**
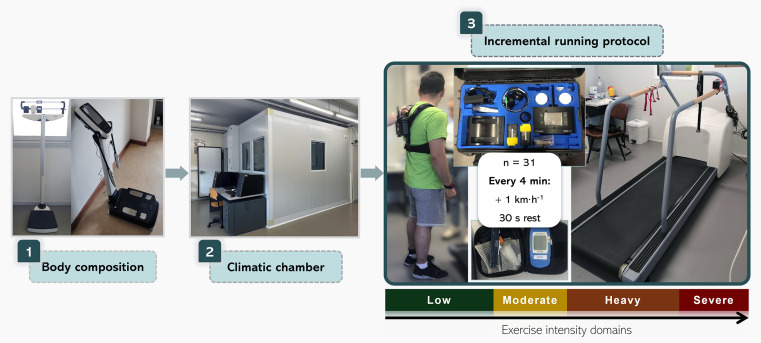
Experimental setup adopted during the data collection.

During the protocol, pulmonary gas exchange variables were measured using a portable telemetric gas analyser (COSMED K5; Rome, Italy) previously calibrated according to the manufacturer instructions (using ambient air against known concentrations [16% O_2_ and 5% CO_2_] and a 3L calibration syringe), placed on the participant’s back near their body centre of mass to limit interferences during running [32,34]. HR was recorded continuously using a chest strap monitor (Garmin Edge 830; Olathe, KS, USA), transmitting data to the gas analyser. Capillary blood samples (5 μL) for lactate concentration analyses were collected from the fingertip using standardised pressure techniques to ensure sampling consistency (Lactate Pro2; Arkay Inc., Kyoto, Japan) at baseline, during the 30 s rest periods and at 1^st^, 3^rd^, 5^th^ and 7^th^ min (until obtaining maximal values) of the recovery phase [33,34] (Fig 2). All 31 participants were included in all analyses, and all variables were obtained and analysed according to the STROBE Statement for cross-sectional research [37].

### Data analysis

The total test duration was 33 ± 4 min, with participants completing 7 ± 1 steps until volitional exhaustion. Collected ventilatory data were filtered to exclude artefacts (e.g., swallowing and coughing), retaining only values within mean ± 3 standard deviations (SD), which were then smoothed using a 10 s moving average [32,34]. An average of 6.57% of data points per participant were filtered, corresponding to 8.57% of all records, with the severe domain having the highest number of excluded points. All participants’ datasets remained usable after this procedure. Next, the mean values from the last 30 s of exercise were used for comparisons, with conventional physiological criteria applied to define the maximal VO_2_: (i) VO_2_ plateau between the last two steps (≤ 2.1 mL·kg^−1^·min^−1^); (ii) blood lactate concentrations ≥ 8 mmol·L^^−^1^; (iii) respiratory exchange ratio ≥ 1.0, used as a conventional supportive criterion for maximal effort verification; (iv) maximal HR > 90% of age-predicted maximal HR; and (v) volitional exhaustion (controlled visually and case-by-case) [32,38]. Age-predicted maximal HR was estimated using the formula: 208 − (0.7 × age) [39]. If a plateau of less than 2.1 mL·kg^−1^·min^−1^ could not be observed, the velocity associated with maximal VO_2_ was calculated as proposed in previous studies [32,40].

The energy expenditure was estimated by counting the contributions from the aerobic and anaerobic lactic sources [32,41], with the former being calculated based on the time integral of net VO_2_ (difference between each step mean value and the baseline value) [26,32]. The anaerobic lactic contribution was estimated based on the net change in [La−] relative to baseline, using the equation: Anaerobic lactic = β [La−] × M, where β represents the O_2_ equivalent (3 mL·kg^^−^1^·mM^^−^1^) and M is the subject body mass (kg) [32,41]. Blood lactate values obtained during the 30 s rest periods following each stage were used to estimate anaerobic contribution for the corresponding stage, and these values were subsequently aggregated according to the predefined exercise-intensity domains (low, moderate, heavy and severe) [33,42]. This approach assumes that lactate measured during the rest interval primarily reflects the metabolic demand of the preceding stage, although it represents the balance between production and clearance [42]. Energy contributions from aerobic and anaerobic systems were quantified in kJ, assuming an energy equivalent of 20.9 kJ·L^^−^1^ [26,32,41].

**Fig 3:**
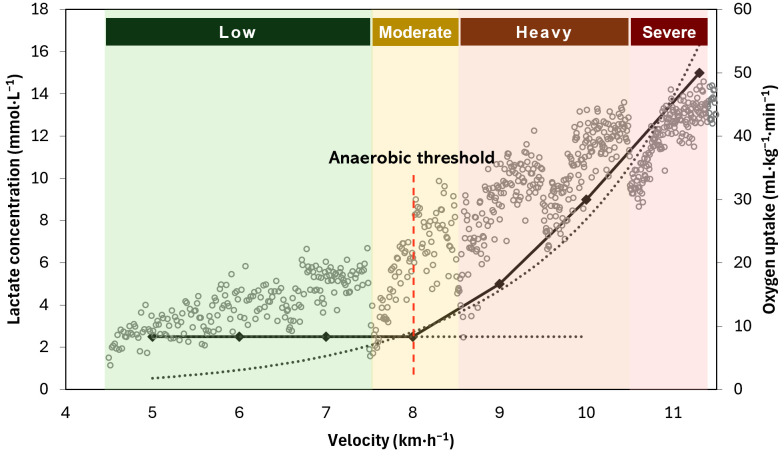
Individual oxygen uptake (circle markers) and blood lactate responses (diamond markers) during the incremental running protocol, allowing determination of low to severe intensity domains. The dashed lines represent the paired exponential–linear regressions whose intersection identifies the anaerobic threshold.

The lactate-velocity curve modelling method was used to identify each subject’s anaerobic threshold by determining the interception point of the best fit of a combined exponential and linear pair of regressions (Fig 3) [33,34]. Using the maximal VO_2_ and the anaerobic threshold as physiological indicators, the low, moderate, heavy (the stages under, at and above the anaerobic threshold, respectively) and severe (the stage where maximal VO_2_ was elicited) exercise intensity domains were established [32,38]. Maximal VO_2_ was examined according to the age-based threshold levels in the NFPA 1580 standard [19], which recommends evaluating cardiorespiratory fitness for firefighters by adjusting for age bands using a treadmill, thereby describing a modality‑independent approach applicable to any valid assessment of maximal VO_2_ [19].

### Statistical analysis

Based on a priori power analysis on G*Power software (version 3.1.9.7; Heinrich-Heine-Universität Düsseldorf, Germany), and assuming a power of 0.80, a medium effect size (f = 0.25), a correlation among repeated measures of 0.5 and an α = 0.05, a sample size of 24 participants was required for conducting the study. All calculations were performed using SPSS 30.0 for Windows (IBM Corp., Armonk, NY, USA), with descriptive statistics presented as mean and SD. Data normality was checked through the Shapiro–Wilk test and one-way repeated-measures ANOVA (with a Bonferroni post hoc test) was applied to compare cardiorespiratory and energetic variables across exercise intensities. Simple linear regression and Pearson correlation were also used to characterise the degree of association between physical fitness and body composition variables. Partial eta-square (η_p_^2^) was computed for effect size calculation, comparing the magnitude of changes throughout intensity domains, considering values of 0.05 − 0.25, 0.25 − 0.64 and ≥ 0.64 as minimum, moderate and strong, respectively. A repeated-measures t-test with Cohen’s d effect size (≥ 0.2, 0.5 and 0.8 as small, medium and large) was used only for pre-specified contrasts (low–moderate; moderate–heavy and heavy–severe) with Holm-Bonferroni correction. Significance level was set at *p* < 0.05.

## Results

**Fig 4:**
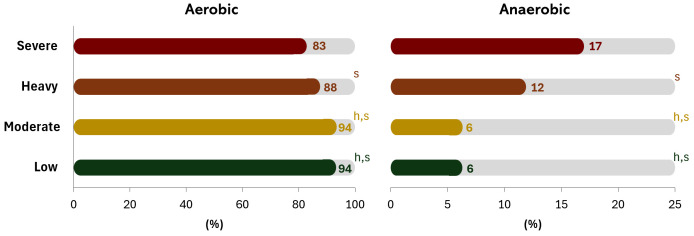
Mean aerobic and anaerobic relative energy contributions (rounded to the closest unit, %) across the four exercise intensity domains. ^h and s^ Different from heavy and severe intensity domains, respectively (*p* < 0.05).

Table 2 details the values of the measured cardiorespiratory variables and energy contributions during each exercise intensity domain. The gradual increment of exercise intensities had a moderate effect in maximal VO_2_, respiratory frequency, HR (bpm), maximal HR (%) and aerobic energy (η_p_^2^ = 0.560, η_p_^2^ = 0.459, η_p_^2^ = 0.548, η_p_^2^ = 0.593 and η_p_^2^ = 0.536, respectively) and a strong effect in ventilation, blood lactate concentrations, anaerobic energy and energy expenditure (η_p_^2^ = 0.676, η_p_^2^ = 0.663, η_p_^2^ = 0.663 and η_p_^2^ = 0.654, respectively), with minimal effect only for respiratory exchange ratio (η_p_^2^ = 0.095). Differences were observed throughout the intensity spectrum, except for the relative aerobic and anaerobic energy, which did not differ between the low and moderate intensity domains (Fig 4). Both absolute and relative aerobic energy contributions exceeded anaerobic outputs across all intensity domains, although anaerobic involvement increased notably at heavy and severe intensities. Between intensity transitions, the largest effect sizes occurred between moderate–heavy intensity domains (d = 1.28–2.22), with smaller differences noted between low–moderate (d = 0.34–0.78) and heavy–severe (d = 0.52–1.19) transitions.

**Table 2:** Mean ± standard deviation values of the cardiorespiratory and bioenergetic variables at low, moderate, heavy and severe running intensity domains.

Variables	Low	Moderate	Heavy	Severe
Oxygen uptake (mL·kg^^−^1^·min^^−^1^)	23.6 ± 7.5 ^m,h,s^	29.1 ± 7.2 ^h,s^	39.9 ± 6.8 ^s^	44.1 ± 7.6
Respiratory frequency (breaths·min^−^^1^)	30.29 ± 9.11 ^m,h,s^	34.10 ± 8.10 ^h,s^	44.20 ± 7.41^s^	50.12 ± 9.46
Ventilation (L·min^^−^1^)	46.00 ± 16.67 ^m,h,s^	59.22 ± 16.75 ^h,s^	96.01 ± 18.42 ^s^	113.60 ± 22.89
Respiratory exchange ratio	0.94 ± 0.13 ^m,h,s^	0.99 ± 0.19 ^h,s^	1.05 ± 0.16	1.07 ± 0.17
Heart rate (bpm)	133 ± 24 ^m,h,s^	150 ± 21 ^h,s^	178 ± 15 ^s^	185 ± 14
Maximal heart rate (%)	72 ± 12 ^m,h,s^	81 ± 10 ^h,s^	96 ± 7 ^s^	100 ± 7
Blood lactate concentration (mmol·L^^−^1^)	3.2 ± 1.3 ^m,h,s^	3.7 ± 1.2 ^h,s^	9.1 ± 3.3 ^s^	12.7 ± 4.3
Anaerobic energy (kJ)	7.3 ± 4.8 ^m,h,s^	9.8 ± 6.3 ^h,s^	30.1 ± 14.7 ^s^	50.0 ± 18.1
Aerobic energy (kJ)	120 ± 48 ^m,h,s^	156 ± 49 ^h,s^	226 ± 47 ^s^	253 ± 53
Energy expenditure (kJ)	127 ± 50 ^m,h,s^	165 ± 51 ^h,s^	256 ± 51 ^s^	303 ± 51

m, h and s Different from moderate, heavy and severe exercise intensities, respectively (*p* < 0.05, determined by one-way repeated-measures ANOVA with a Bonferroni post hoc test).

**Fig 5:**
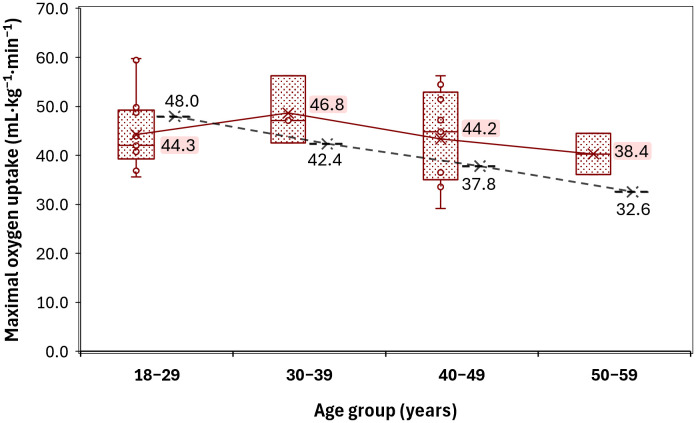
Distribution of maximal oxygen uptake across age groups, presented as box-and-whisker plots. Individual observations are shown as dark red circles, while group means are indicated by overlaid trend lines and corresponding numerical labels in shaded red. NFPA 1580 age-specific thresholds are depicted by black dashed lines and markers, with values annotated for each age group.

The number and percentage of participants meeting each VO_2max_ verification criterion were as follows: VO_2_ plateau, n = 26 (84%); blood lactate ≥ 8 mmol·L^−1^, n = 31 (100%); respiratory exchange ratio (RER) ≥ 1.0, n = 25 (81%); HR ≥ 90% of age-predicted maximum, n = 28 (90%); and volitional exhaustion, n = 31 (100%). The VO_2_ age-specific compliance with the NFPA 1580 thresholds is described in Table 3, which addresses, for each age band, the threshold used, the number of participants in that band and the percentage meeting and not meeting the threshold. In addition, intergroup variability is illustrated in Fig 5. Maximal VO_2_ values stratified by age ranged from 38.4–46.8 mL·kg^-^−^1^·min^^−^1^, with the lowest values observed in the youngest group (18–29 years). Overall, maximal VO_2_ distribution was: > 50.0 mL·kg^-^−^1^·min^^−^1^ (n = 6), 40.0–49.9 mL·kg^^−^1^·min^^−^1^ (n = 16) and 33.0–39.9 mL·kg^^−^1^·min^^−^1^ (n = 9).

**Table 3:** Mean ± standard deviation values of maximal oxygen uptake (VO_2max_) and compliance verification with each age band addressed by the NFPA standard.

Age	NFPA threshold (mL·kg ^− 1^·min ^− 1^)	n	VO_2max_ (mL·kg ^− 1^·min ^− 1^)	n (%) Compliant	n (%) Not compliant
18 − 29	48.0	17	44.3 ± 8.4	5 (29)	12 (71)
30 − 39	42.4	3	46.8 ± 11.5	3 (100)	0 (0)
40 − 49	37.8	9	44.2 ± 6.3	5 (56)	4 (44)
50 − 59	32.6	2	38.4 ± 3.9	2 (100)	0 (0)

**Fig 6:**
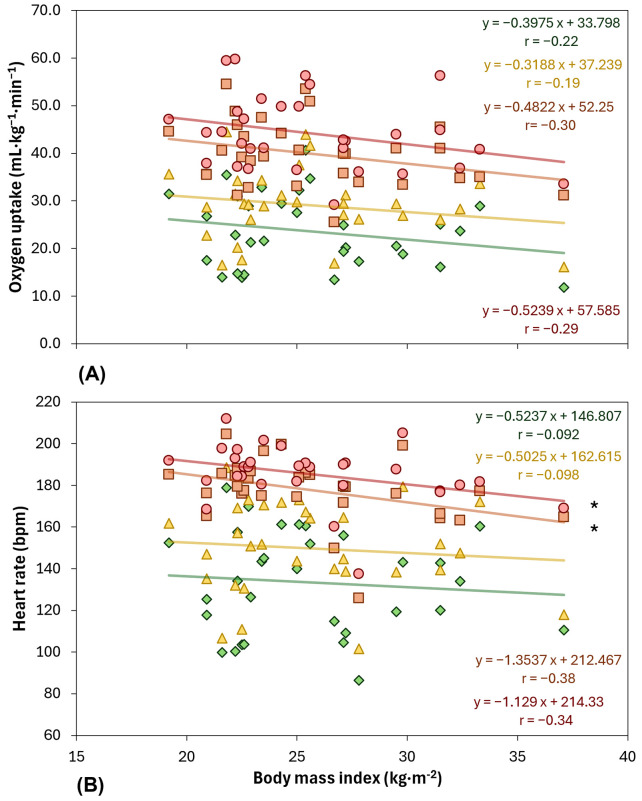
Oxygen uptake and heart rate plotted against body mass index across low (green), moderate (yellow), heavy (orange) and severe (red) intensity domains. Regression equations and corresponding correlation coefficients are provided for each domain. * Differences between steps (*p* < 0.05).

**Fig 7:**
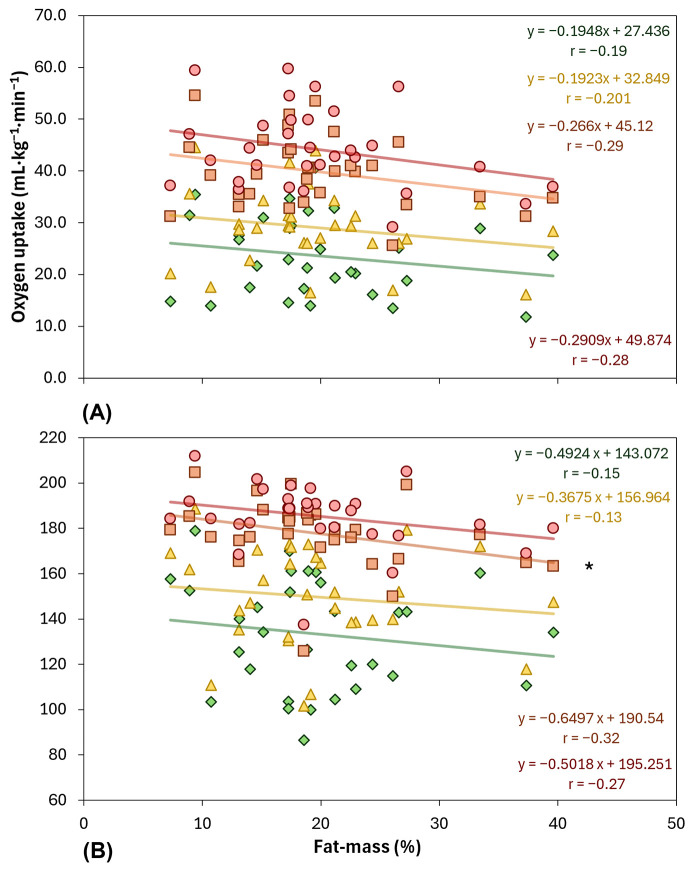
Oxygen uptake and heart rate plotted against fat-mass percentage across low (green), moderate (yellow), heavy (orange) and severe (red) intensity domains. Regression equations and corresponding correlation coefficients are provided for each domain. * Differences between steps (*p* < 0.05).

The associations of VO_2_, HR with body mass index and fat mass are displayed in Figs 6 and 7, with low negative associations being found between HR and body mass index at heavy (r = −0.38, *p* = 0.017) and severe (r = −0.34, *p* = 0.028) intensities, and with fat mass at heavy intensity (r = −0.32, *p* = 0.037).

## Discussion

The current study characterised the physiological profile of male volunteer firefighters by examining their cardiorespiratory fitness and energy systems contributions during incremental running exercise tailored to individual capacity. The overall maximal VO_2_ (~44 mL·kg^−1^·min^−1^) exceeded the general reference value of ~42 mL·kg^−1^·min^−1^ [18] but, considering the age-based recommendations [19], ~ 52% of participants did not reach the threshold limits. Most participants between 18 − 29 years had lower maximal VO_2_ values than the minimum recommended (~44 vs. ~ 48 mL·kg^−1^·min^−1^), while aged firefighters were more likely to meet the age-based thresholds. There was an increase in the anaerobic system contribution during the heavy and severe intensity domains, although the aerobic energy system accounted for ~88% and ~83% of the total demand in these two intensities (respectively). Moreover, the hypothesis that firefighters’ cardiorespiratory fitness and body composition variables (i.e., body mass index and fat mass) would display significant associations was only partially confirmed in the heavy and severe intensity domains.

Maximal VO_2_ is the gold standard measure of physical fitness and a key indicator of cardiovascular health [4,43]. The maximal VO_2_ values observed (~44 mL·kg^^−^1^·min^^−^1^) exceeded the general minimum criterion for occupational activities (~42 mL·kg^−1^·min^−1^) and previous results reported in volunteer firefighters (~39 mL·kg^^−^1^·min^^−^1^) [21,29]. However, their maximal VO_2_ remained below that reported for career firefighters (~45 − 54 mL·kg^^−^1^·min^^−^1^) [11,20,44], likely reflecting reduced training opportunities among volunteers despite their equally demanding tasks [21,22]. Considering other tactical populations, volunteer firefighters exhibit intermediate maximal VO_2_ values, lower than those of military personnel and higher than those of law enforcement officers [6,45]. In our sample, only 15 participants (~48%) met the NFPA 1580 age-based thresholds [19], with the youngest group (18 − 29 years) showing the lowest compliance (~29%), which is physiologically unusual. A plausible explanation is that this result reflects sample-specific characteristics (e.g., differences in training status, lack of experience with physically demanding activities, or other unmeasured confounders) and should be interpreted cautiously, not as an age-related physiological trend. The highest maximal VO_2_ values were seen among those aged 30 − 39 years (~47 mL·kg^^−^1^·min^^−^1^), consistent with previous studies [20].

Studies of simulated and live firefighting tasks described VO_2_ values of 30 − 45 mL·kg^^−^1^·min^^−^1^ as the minimum VO_2_ required to perform these activities safely [9,16,43]. Since a higher maximal VO_2_ reduces the relative intensity of the task, firefighters with lower maximal VO_2_ must work closer to their maximum capacity to complete strenuous activities [3,5,46]. The anaerobic threshold, defined as the boundary at which lactate steady-state behaviour changes, is a key cardiovascular and metabolic indicator [26,47,48]. Participants reached this boundary at ~66% of their maximal VO_2_ (~29 mL·kg^−1^·min^−1^), after which a progressive physiological perturbation was observed, as reflected by increases in ventilation, respiration exchange ratio and blood lactate. As expected, minute ventilation, respiratory frequency, and respiratory exchange ratio increased progressively throughout the exercise, indicating higher ventilatory and metabolic demands with rising intensity [33]. The elevation in these variables reflects greater breathing effort and a shift toward carbohydrate metabolism as exercise approaches exhaustion [25]. Since the anaerobic threshold corresponds to the upper limit of the moderate intensity domain, the results suggest that firefighters may often perform tasks above this level, increasing their physiological load and accelerating fatigue [14,46]. Nevertheless, despite its relevance, anaerobic power and capacity remain understudied among firefighter populations [24,47]. Moreover, this laboratory-based treadmill assessment was conducted without protective equipment or thermal stress, potentially underestimating the demands of firefighting.

Maximal HR (%) increased progressively to ~100%, similar to values observed during live emergencies (81–97% of maximal HR have been observed during fire suppression duties [9,23] and other tactical scenarios [3,37]). Due to the high cardiovascular strain and additional occupational stressors such as shift work, sleep deprivation and poor diet [2], firefighters face an elevated risk of cardiovascular disease, highlighting the importance of continuous fitness monitoring and prevention programs [4]. Furthermore, high blood lactate concentrations of up to ~13 mmol·L^−1^ were recorded at the end of the severe intensity, equivalent to previously reported values during firefighting tasks [49] and tactical training [50]. While elevated lactate values are a natural response to intense activity, they reflect high-intensity metabolic demand. They may co-occur with increased cardiac output, pulmonary ventilation and metabolic demands, as well as with physiological disturbances that contribute to fatigue and reduced performance [51,52]. These findings suggest that adequate fitness levels may enable firefighters to maintain their working performance and reduce the risk of overexertion injuries [49]. In addition, the strong effect of the exercise intensities on ventilation reinforces its importance in improving exercise tolerance and decreasing physical exertion [53], which are also determinants of other occupational risks (e.g., the potential inhaled dose of air pollutants during fires [54]).

The absolute and relative energy contribution values displayed higher contributions from the aerobic system, like studies conducted among other occupational populations [1,27,32]. Correspondingly, firefighting has been characterised as a highly aerobic occupation, in which up to 86% of the energy contributions are supported by this system [24,49], demonstrating that results can help describe the potential energy demands of their working duties in a controlled environment. Nevertheless, in addition to the strong demand of the aerobic system, the high lactate values at the end of the protocol reflect the important contribution of anaerobic energy sources to energy supply [46]. Depending on the severity of an emergency, the duration of firefighting tasks can range from minutes (e.g., stair climbing or forcible entry) to hours, days or weeks [12]. Therefore, firefighter tasks can depend on all body energy systems, with their intensity and duration often dictating which energy system is primarily used [24,55]. Consequently, training programs should target improvements in both energy systems through combined aerobic and strength training to meet the high physiological demands of the profession [24]. Because the present test included brief rest intervals for lactate sampling, direct comparisons with occupational thresholds derived from continuous protocols should be interpreted cautiously.

Although relationships between cardiorespiratory fitness and body composition have been previously examined in firefighters [11,44], they remain largely unexplored across different exercise intensity domains in volunteer populations. A low negative association was found between HR and both body mass index and fat mass percentage (r <−0.32). No significant correlations were found between maximal VO_2_ and the assessed body composition variables (i.e., body mass index and fat mass percentage), differing from previous studies that reported stronger associations [11,16,56]. These trends are similar to other first responder studies, suggesting that other factors (e.g., optimised energy intake, adequate sleep and strength training) can improve body composition in volunteer firefighters [28,57]. Therefore, health-promoting fitness programs for volunteer firefighters should also incorporate factors such as nutritional guidance, stress management and sleep hygiene education [56].

The NFPA does not prescribe a mandatory protocol for maximal VO_2_ testing; nevertheless, the applied intermittent incremental protocol used in this study complies with physiological testing standards [27,32,33]. While physical fitness has been previously evaluated among other first responder populations [3,15,27,28] and several incremental exercise protocols have been applied to assess firefighter cardiorespiratory fitness [30,31,58], the present protocol proved more suitable for individual characterisation. It was specifically designed to identify key physiological markers across multiple intensity domains [33,34]. Although firefighters’ physiological and bioenergetic variables have been previously examined [7,31], integrating these variables into a treadmill protocol adjusted to each volunteer’s capacity has not yet been addressed [2,15]. Since we observed differences with moderate to strong effect sizes in most variables while fulfilling physiological criteria for maximal VO_2_ determination [38], we can confirm the adequacy and usefulness of our protocol among volunteer firefighters**.**

Some limitations of the current study should be acknowledged. Although the protocol effectively assessed cardiorespiratory and bioenergetic variables, it was conducted in thermoneutral environmental conditions. Physical and thermal loads were not included since they would have accelerated the transition to exhaustion. Nevertheless, firefighters must wear protective gear and work under adverse climatic conditions during their occupational activities [8,23]. Future research should examine these responses under controlled thermal conditions and real firefighting scenarios. Participants self-reported performing ≥ 3 sessions per week of vigorous activity, but no training logs were assessed, limiting monitoring of fitness-related variables beyond maximal VO_2_. Despite the sample being comparable to previous literature [11,58,59], future studies should include larger and more diverse samples, involving both sexes, to verify compliance with NFPA criteria [19]. Since the sample focused on male, physically active volunteers from two fire brigades, the external validity to other brigades, less active volunteers and female firefighters is uncertain. In addition, the estimation of anaerobic energy was limited to the lactic component, as the alactic contribution was not included, which may have led to a slight underestimation of total energy expenditure (aerobic, anaerobic lactic and anaerobic alactic energy contributions) across exercise intensity domains. As the NFPA standards currently lack muscular strength assessments, their inclusion is recommended for future works, given their critical relevance to firefighting tasks (e.g., lifting victims, dragging hoses and climbing ladders).

### Practical applications

The findings of this study indicate that most volunteer firefighters did not meet the minimum recommended maximal VO_2_ levels for safe occupational performance, aligning with previous research highlighting insufficient cardiorespiratory fitness in firefighter populations [5,16,21]. Interventions should be prioritised for firefighters below age-specific NFPA thresholds, with training intensity and progression tailored to baseline fitness and injury risk. To address this deficiency, the literature supports the implementation of high-intensity interval training (HIIT) as an effective strategy to enhance cardiovascular efficiency, VO_2_ and overall work capacity among firefighters and other tactical professionals [13,50,60]. Workplace-based HIIT protocols, consisting of 4 x 4 min bouts at ≥ 90% of maximal VO_2_ interspersed with 4 min recovery periods, have been shown to improve cardiorespiratory fitness and occupational performance with reduced time commitment [13,61]. In addition, effective training can be performed with either minimal or maximal access to equipment facilities [60], allowing immediate implementation in fire brigade settings. Complementarily, strength and endurance training are also recommended, as they are fundamentally related to improvements in cardiorespiratory capacity [10,23,62].

## Conclusion

The current study offers insights into the physiological profile of male volunteer firefighters using an incremental running exercise protocol adapted to individual capacity and performed under controlled conditions, demonstrating adjustments in physiological responses as exercise intensity increases until exhaustion. Slightly more than half of participants (16 of 31; ~ 52%) exhibited below-recommended age-based cardiorespiratory fitness, which may impair performance and health. Although the outcomes reported in this study are specific to firefighters, the approach to cardiorespiratory fitness assessment and characterisation can be directly applied to other occupational training environments, including those of first responders and tactical professions.
